# Distribution of mutational fitness effects and of epistasis in the 5’ untranslated region of a plant RNA virus

**DOI:** 10.1186/s12862-015-0555-2

**Published:** 2015-12-07

**Authors:** Guillermo P. Bernet, Santiago F. Elena

**Affiliations:** Instituto de Biología Molecular y Celular de Plantas, Consejo Superior de Investigaciones Científicas-UPV, Campus UPV CPI 8E, Ingeniero Fausto Elio s/n, 46022 València, Spain; The Santa Fe Institute, 1399 Hyde Park Road, Santa Fe, NM 87501 USA

**Keywords:** Distribution of mutational fitness effects, Plant virus, Potyvirus, RNA regulatory sequences, RNA folding, Virus evolution

## Abstract

**Background:**

Understanding the causes and consequences of phenotypic variability is a central topic of evolutionary biology. Mutations within non-coding *cis*-regulatory regions are thought to be of major effect since they affect the expression of downstream genes. To address the evolutionary potential of mutations affecting such regions in RNA viruses, we explored the fitness properties of mutations affecting the 5’-untranslated region (UTR) of a prototypical member of the picorna-like superfamily, *Tobacco etch virus* (TEV). This 5’ UTR acts as an internal ribosomal entry site (IRES) and is essential for expression of all viral genes.

**Results:**

We determined in vitro the folding of 5’ UTR using the selective 2’-hydroxyl acylation analyzed by primer extension (SHAPE) technique. Then, we created a collection of single-nucleotide substitutions on this region and evaluated the statistical properties of their fitness effects in vivo. We found that, compared to random mutations affecting coding sequences, mutations at the 5’ UTR were of weaker effect. We also created double mutants by combining pairs of these single mutations and found variation in the magnitude and sign of epistatic interactions, with an enrichment of cases of positive epistasis. A correlation exists between the magnitude of fitness effects and the size of the perturbation made in the RNA folding structure, suggesting that the larger the departure from the predicted fold, the more negative impact in viral fitness.

**Conclusions:**

Evidence that mutational fitness effects on the short 5’ UTR regulatory sequence of TEV are weaker than those affecting its coding sequences have been found. Epistasis among pairs of mutations on the 5’ UTR ranged between the extreme cases of synthetic lethal and compensatory. A plausible hypothesis to explain all these observations is that the interaction between the 5’ UTR and the host translational machinery was shaped by natural selection to be robust to mutations, thus ensuring the homeostatic expression of viral genes even at high mutation rates.

## Background

Viruses have to face many different challenges along their life cycle. Successful infections require viruses to evade host defenses and use cellular resources and machinery to produce new virions. To maximize their fitness under a hostile environment, virus genomes must necessarily be able to adapt and evolve. Since Jacob and Monod [1] postulated the mechanistic basis for gene regulation, the importance of gene regulation in evolution has been recognized. It was postulated that the fitness of microorganisms should rely on two aspects of gene regulation, the coordinated induction of functionally related genes and the repression of unnecessary ones. Concerning viruses, the evolution of gene regulation must also be fundamental, as diverse strategies for gene expression regulation have been implemented on viruses that differ in the nature of their genomes.

Some viruses encode polymerases for autonomous genome replication and/or transcription while others take profit of host enzymes to do so, but viruses do not encode for mRNA translation machinery, and consequently ribosomes and other molecules essential for viral biosynthesis have to be supplied by the host cell [2, 3]. Initiation of protein synthesis is almost entirely dependent on the translational system of infected cells, and propagation of viral genomes will mostly rely on their capacity to somehow control and effectively compete with the host for translational resources. Translation of mRNAs in eukaryotes is a complex process subject to diverse regulatory controls [4]. In brief, cellular mRNAs present a methylated 5’ cap structure (m^7^GpppN) that is recognized by eukaryotic initiation factors ultimately leading to the recruitment of ribosomes and the initiation of protein synthesis. Recognition of the 5’ cap on mRNAs is a key step in cellular regulation of translation, and viruses have evolved a profusion of non-canonical strategies able to bypass host regulatory mechanisms thus allowing translation to be tailored to their needs [5]. One of such non-canonical strategies is a cap-independent mechanism of translation initiation in which the ribosome is guided to the initiation codon by structural motifs (internal ribosome entry site, IRES) in the 5’-untranslated region (5’ UTR) of the mRNA. IRES-mediated translation is common among RNA viruses, but few similarities in size, sequence or structure can be found among IRES in different families of viruses. IRES elements in animal RNA virus are usually long and highly structured, while those in plants are smaller and less structured [6]. Besides enhancement of protein translation, 5’ UTR sequences of viral genomes are involved in regulation of genome replication [7–13]. Viral 5’ UTRs are non-coding regulatory regions with potential roles in almost every step of viruses’ life cycle and certainly relevant for viral fitness.

Several studies have characterized the fitness effects of mutations in model cellular organisms [14–16] and viruses [17–20]. The distribution of mutational fitness effects (DMFE), that is, the fraction of all possible mutations that are beneficial, neutral or deleterious, is pivotal for evolutionary biology and can provide insights into functional synthesis studies connecting changes in gene sequence to changes in phenotype and fitness [21]. The DMFE is highly variable between species or genomic regions and also highly dependent on environmental conditions [22, 23] but, as a general trend, advantageous mutations leading to increased fitness are rare and sensitivity to mutation is higher in viral genomes compared to that of more complex cellular genomes [24–27]. RNA viruses are ideal systems for characterizing mutational fitness effects and the nature of interactions among mutations (epistasis); small compact genomes capable of folding to functional RNA secondary structures and encoding for multifunctional proteins are expected to be not only very sensitive to mutation but also prone to strong epistasis. Indeed, magnitude epistasis (ME) is reported to be common for diverse RNA viruses [28, 29], i.e. the fitness effect associated with a mutation, but not its sign, vary depending on the viral genetic background. However, most DMFE and quantitative epistasis studies have been focused on coding sequences and relatively little is known about the mutational effects and putative epistatic interactions in non protein-coding regulatory sequences. To the extent of our knowledge, only two studies have previously characterized mutational fitness effects and epistasis in viral regulatory regions. On the one hand, the region U5-IR of *Rous sarcoma virus* (RSV) showed extremely deleterious fitness effects and, on average, positive epistasis [30], that is to say, mutations had smaller effect in combination than alone. On the other hand, for the transcriptional promoter of *Human immunodeficiency virus* type 1 (HIV-1), deleterious and beneficial mutations were reported to occur at high and similar frequencies but no significant epistasis among mutations was found [31].

**Fig. 1:**
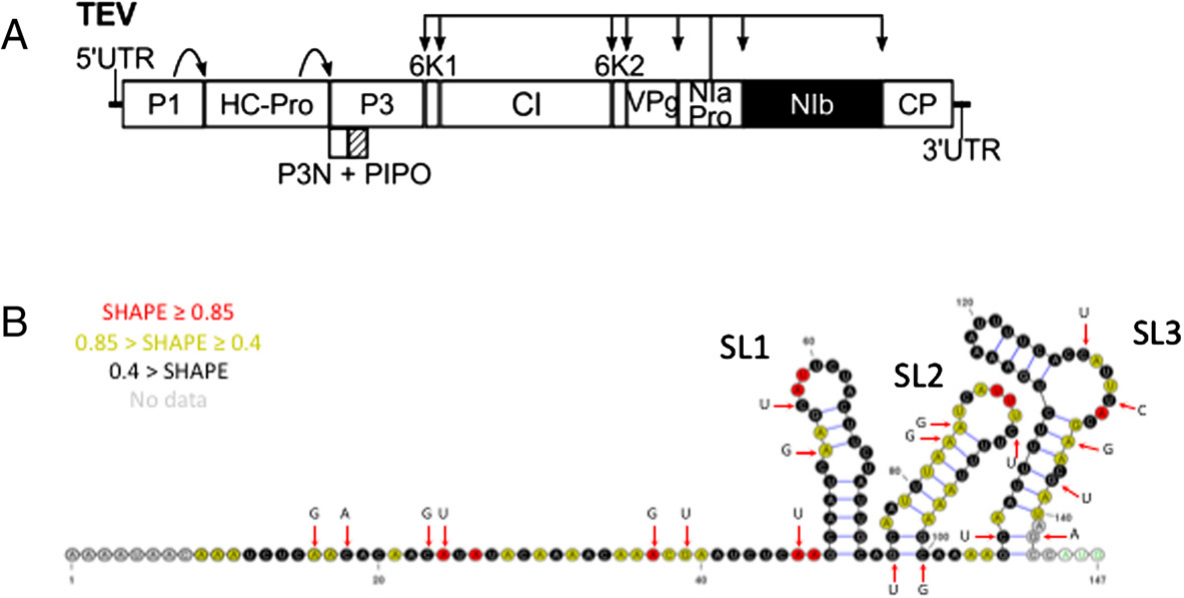
**a** Schematic representation of TEV genome, indicating the two non-coding regions and the 11 cistrons. The arrows indicate the proteolytic positions targeted by the corresponding proteases (P1, HC-Pro and NIaPro). **b** Minimum free energy structure for the WT TEV 5’ UTR. SHAPE probing data (reactivity) is represented with red, yellow or black colors at each nucleotide position. Red arrows point to nucleotides that have been mutagenized in this study, with indication of the mutant nucleotide. SL1, SL2 and SL3 indicate the three stem-loops defining the IRES

In this study we aim to characterize the fitness effects, and spectrum of epistasis for mutations at the 5’ UTR sequence of *Tobacco etch virus* (TEV; genus *Potyvirus*, family *Potyviridae*), a plant-infecting virus. *Potyvirus* are members of the picorna-like superfamily of positive-sense RNA viruses. Their genomic organization is highly similar to animal picornaviruses in that the genomic RNA functions as a monocistronic mRNA encoding a single polyprotein of about 10 kilobases that, upon synthesis, is self-processed by virus-encoded proteases into all the mature peptides. The TEV genome codifies 11 proteins, 10 of them transcribed from a single cistron [32] and the other one synthesized by translational frameshift [33] (Fig. 1a). The TEV 5’ end is covalently linked to a virus-encoded protein (VPg) and has a polyA tail at the 3’ end. The TEV 5’ UTR spans 144 nucleotides and contains IRES activity [34, 35], with two non-redundant hypothetical CIREs (cap-independent regulatory elements) [36, 37] likely responsible for promoting cap-independent translation in cooperation with the virus polyA tail [38, 39]. Although picornavirus IRES are typically large and highly structured, the TEV element is rather small, representing one of the most compact viral elements identified that can promote cap-independent translation [35, 38, 39]. The precise TEV 5’ UTR sequences and folding structure required for enhancing TEV translation remain uncertain but it is to be expected that mutations in such regions will have significant impact on viral fitness. The DMFE and the spectrum of epistasis have been characterized for the TEV ORF. Random nucleotide substitutions affecting TEV coding cistrons have been reported to reduce fitness by nearly 50 % on average, with up to 40 % of mutations being lethal [19]. Similarly, the DMFE of mutations for TEV coding regions was described to be left-skewed (i.e. containing more negative effects than expected in a Gaussian distribution) and leptokurtic (i.e. comprising less central values and having heavier tails) [19], while, on average, positive epistasis is significantly more abundant, and takes the form of sign epistasis (SE) [29].

To achieve our goals, we first evaluated the secondary structure of TEV 5’ UTR *in silico* and in vitro using the selective 2’-hydroxyl acylation analyzed by primer extension (SHAPE) technique. Second, we created a collection of single-nucleotide substitution mutants in the 5’ UTR of TEV and quantified their fitness effects in the context of the predicted secondary structure. Third, we created a collection of double mutants by combining mutations of known fitness effects and evaluated the intensity and nature of epistasis. Finally, we compared all these results with those previously reported for random mutations affecting the protein-coding sequence of TEV.

## Methods

### Plants and virus

TEV fitness effects were evaluated on *Nicotiana tabaccum* L. cv. Xanthi NN plants (Lehle seeds), known to be natural hosts for the virus [40]. The pMTEV plasmid is a transcription vector that includes the complete wild-type (WT) TEV genome (GenBank accession DQ986288) [41] and was used as a source for TEV sequences whenever they were required along the experiments here referred.

### Analysis of TEV 5’ UTR folding

All possible single mutants at TEV 5’ UTR sequence were generated *in silico* and computed to obtain their minimum free energy secondary structure (MFESS) using the RNAfold program from the ViennaRNA package version 1.6.4. [42] and the LocARNA webserver [43]. The structures generated by both algorithms were identical. The structural robustness of each mutant was then evaluated as described in Sanjuán et al. [44]. In short, the RNAdistance program (also from the ViennaRNA package; default parameter configuration) was used to compare the predicted secondary structure of each single mutant with that of the WT, and the resulting Hamming distance between the brackets-and-dots representation of structures, *d*_*ij*_, was then scaled to the 5’ UTR length (*L* = 144) to get a measure of the mutational effect *s*(*d*_*ij*_) = *d*_*ij*_/*L*. The smaller the *s*(*d*_*ij*_) value, the less effect a mutation has on the folding. Sites to be mutagenized at TEV 5’ UTR were selected according to the results obtained in this *in silico* analysis. The MFESS for the WT TEV 5’ UTR sequence, computed at 25 °C was −13.1 kcal/mol. 146 out of all 432 possible single mutants would not affect the expected WT secondary structure. For the other 286 possible single mutants, the *in silico* predicted MFESS values were affected to a variable extent, indicating a relative weak structural robustness at the evaluated region [44, 45]. Thus, five structurally neutral mutations (notice that structurally neutral does not necessarily means biologically neutral), five mutations whose structural effects are weak (in the 1^st^ quartile of the distribution of *s*(*d*_*ij*_) values), five mutations of moderate structural effect (between the 1^st^ and the 3^rd^ quartiles), and five mutations of large structural effect (in the 4^th^ quartile) were selected. This adds up to a total of 20 mutations. Another subset of 25 double mutants was randomly chosen from combinations of this 20 single mutants.

To better characterize the native secondary structure of the TEV 5’ UTR, the purified in vitro transcribed 5’ UTR RNA (see below) was subjected to SHAPE [46, 47] resolved in two capillaries [47]. In brief, 2 μg of the 5’ UTR RNA were folded (100 mM HEPES, 6 mM MgCl_2_, 100 mM NaCl), modified with the SHAPE reagent (incubation with N-methylisatoic anhydride (NMIA) 6 mM for at 37 °C for 35 min) and purified through ethanol precipitation. Primer extension reactions with 4 pmoles of fluorescently VIC- and NED-labeled versions of primer 5’-GACTGTGCCAAAGATGAGTGCCATG-3’ were then performed and the resulting cDNAs analyzed by capillary electrophoresis. Raw electrophoretogram traces were processed with QuShape software [48] to obtain normalized SHAPE reactivities that were implemented in the ShapeKnots module of RNAstructure version 5.7 thus allowing pseudoknot prediction [49]. The resulting secondary structure prediction was obtained from the mean data of four independent SHAPE experiments and visualized with VARNA [50] (Fig. 1b).

### Mutagenesis, inoculation and TEV quantification

The selected mutant genotypes at TEV 5’ UTR were generated by site-directed mutagenesis of pMTEV using QuikChange^®^ II XL Site-Directed Mutagenesis Kit (Agilent Technologies) which incorporates *PfuUltra*™ high fidelity DNA polymerase that minimizes the introduction of undesired mutations. PCR conditions for mutagenesis consisted of an initial 2 min denaturation step at 95 °C followed by 19 cycles of 50 s at 95 °C, 50 s at 60 °C and 13 min at 68 °C, and a final 7 min elongation step at 68 °C. Mutagenic primers were designed according to manufacturer recommendations and the uniqueness of each mutation was later confirmed by sequencing an 800 bp fragment encompassing the mutated nucleotide. After *Bgl*II linearization and purification of successfully mutagenized plasmids, infectious RNAs of each genotype (including WT, single and double mutants) were obtained by in vitro transcription using SP6 mMESSAGE mMACHINE Kit (Ambion) following manufacturer instructions.

Two different inoculation experiments were performed separated by 5 months and differing in the quantity of inoculum per plant (2 μg and 5 μg of RNA transcripts, respectively). In each experiment, five 4-week old *N. tabacum* plants per genotype were rub-inoculated in the third true leaf with 5 μl of viral transcript containing 10 % Carborundum. Control plants were mock-inoculated in the same way with water instead of viral transcripts. All plants were maintained in the greenhouse at 25 °C and 16 h light/day. Fourteen days post-inoculation (dpi), all non-inoculated leaves from each plant were collected and pooled in plastic bags and stored at −20 °C until total RNA extraction by means of the InviTrap Spin Plant RNA Mini Kit (Invitek). Both symptomatic and asymptomatic plants were processed and virus accumulation was measured by absolute RT-qPCR using external standard curves consisting in seven serial dilutions of TEV RNA in vitro transcripts diluted in 100 ng RNA obtained from the mock-inoculated (healthy) plants. Real-time PCR reactions were performed in triplicate in 20 μl reaction volumes using One Step SYBR PrimeScript RT-PCR Kit II (TaKaRa) according to manufacturer instructions and carried out in the Step One Plus Real-Time PCR thermocycler (Applied Biosystems, USA). The primers used (forward, 5´-TTGGTCTTGATGGCAACGTG; reverse, 5´-TGTGCCGTTCAGTGTCTTCCT) target the TEV *CP* cistron thus allowing quantification of TEV complete genomes [22] (Fig. 1a). The thermal profile consisted of an RT stage of 5 min at 42 °C and 10 s at 95 °C, followed by a PCR phase of 40 cycles of 5 s at 95 °C and 34 s at 60 °C, and a final dissociation curve protocol of 15 s at 95 °C, 1 min at 60 °C and 15 s at 95 °C. Amplification results were analyzed with StepOne Software v2.2.2 (Applied Biosystems).

### Measuring fitness and epistasis

Virus accumulation, *Q*_*t*_ (pg of TEV RNA per 100 ng of total plant RNA), was quantified *t* = 14 dpi for the mutant and wildtype viruses by RT-qPCR [51]. A Malthusian growth rate per day (*m*) was computed according to the expression $m=\frac{1}{t} \ln {Q}_t$. Relative fitness (*W*) was then calculated as $W={e}^{m-{\overline{m}}_{WT}}$, where ${\overline{m}}_{WT}$ is the grand mean Malthusian value estimated for WT TEV on the corresponding experimental block.

An epistasis coefficient among pairs of mutations *i* and *j*, *ε*_*ij*_, was calculated as *ε*_*ij*_ = *W*_00_*W*_*ij*_ − *W*_*i*0_*W*_0*j*_ [52], where *W*_00_, *W*_*ij*_, *W*_*i*0_, and *W*_0*j*_ correspond to the relative fitness of the WT, the double mutant and each single mutant, respectively. A value of *ε*_*ij*_ > 0 corresponds to the case of positive epistasis, whereas a value of *ε*_*ij*_ < 0 indicates negative epistasis. Values of *ε*_*ij*_ not significantly departing from zero were qualified as non-epistatic mutational effects. In those cases for which epistasis turned out to be significant, we further proceeded to determine its type: magnitude, sign or reciprocal sign. To do so, we used the inequalities derived by Poelwijj et al. [53]. Magnitude epistasis (ME) occurs when the fitness value associated to a mutation, but not its sign, changes upon the genetic background wherein it appears. ME can be positive or negative, depending on whether the fitness of the double mutant is larger or smaller than expected under the null model. Sign epistasis (SE) refers to cases in which the sign of the fitness effect of a mutation is under epistatic control; thus, such a mutation is beneficial in some genetic backgrounds and deleterious in others [54]. A particular case of SE, know as reciprocal sign epistasis (RSE), occurs when the sign of the fitness effect of a mutation is conditional upon the state of another locus and vice versa [53]. RSE has been shown to be a necessary condition for an adaptive landscape to be rugged [53].

All statistical tests were performed using IBM SPSS version 20 (Armonk, NY, USA). In all cases, reported error intervals correspond to ±1 standard error of the mean (SEM).

## Results

### Description of the RNA structure determined by SHAPE

The SHAPE reagents are relatively insensitive to base identity but very sensitive to conformational dynamics [46]. They preferentially react with flexible nucleotides but poorly with base-pared and protected nucleotides. Therefore, low values of normalized reactivities (Fig. 1b) suggest that nucleotides are involved in base-pairing while high values indicate unprotected nucleotides. Combining SHAPE in vitro reactivity data with *in silico* secondary structure predictions (RNAfold and LocARNA) results in a more accurate representation of TEV 5’ UTR secondary structure (Fig. 1b). This structure consists of a 5’ proximal unstructured region of 47 nucleotides and three distal stem-loops (SL1 to SL3). SHAPE reactivities suggest, however, that some of the nucleotides in this unstructured region are not fully accessible, meaning that they may be somehow protected from the chemicals. SL1 (nucleotides 48–73) includes an asymmetric internal loop while the two others (nucleotides 75–100 and 105–143) contain one and two bulges respectively and an additional mismatch in the case of the SL closer to the initiation codon. No pseudoknot was predicted. The main difference between the *in silico* predicted secondary structure for TEV 5’ UTR with and without SHAPE data lies in the folding of SL3 (data not shown) and thus it can be argued that including SHAPE reactivities helped in discriminating the more reliable *in silico* secondary structure prediction, but no great folding differences were revealed.

### Statistical exploration of the fitness data

The fitness of all mutants was determined in two independent experimental blocks, with a median of five infected plants per block and three technical replicates of the RT-qPCR per plant. Fitness data (Table 1) were fitted to a general linear model (GLM) with a Normal distribution and an identity link function. The model incorporates three random factors: the mutant genotype (*M*) and the experimental block (*B*), which are orthogonal, and the plant replicate (*P*), which is nested within the *M* × *B* interaction term: *W*_*ijkl*_ = *μ* + *M*_*i*_ + *B*_*j*_ + (*M* × *B*)_*ij*_ + *P*(*M* × *B*)_*ijk*_ + *ξ*_*ijkl*_, where *μ* is the grand mean value and *ξ*_*ijkl*_ is the error associated with individual measure *l* (estimated from the technical replicates of the RT-qPCR reaction). The statistical significance of each factor was evaluated using a likelihood ratio test (LRT) that asymptotically follows a *χ*^2^ distribution (Table 2). All factors were significant, indicating heterogeneity among plants and experimental blocks. Despite this experimental noise, the differences among genotypes in fitness were still highly significant. Indeed, to evaluate the magnitude of the different effects included in the model, we used the *η*_*P*_^2^ statistic that represents the proportion of total variability attributable to a given factor. Conventionally, values *η*_*P*_^2^ < 0.05 are considered as small, 0.05 ≤ *η*_*P*_^2^ < 0.15 as medium and *η*_*P*_^2^ ≥ 0.15 as large effects. The factor mutant genotype contributed very significantly to the observed variability in fitness (Table 2). Average fitness values, and their corresponding SEM, were estimated for each genotype from the fitted model. These estimated fitness values will be used in the analyses described in the following sections.

**Table 1:** Data summary. The relative fitness, number of fitness determinations, epistasis coefficient, and the Hamming distance to the WT folding obtained with the RNAdistance program are shown for each genotype. Errors represent ±1 SEM

Mutant genotype	Relative fitness	Number	Epistasis	Hamming distance
WT	1.000 ± 0.006	15		
A16G	0.918 ± 0.033	24		66
C18A	0.958 ± 0.042	12		0
C23G	0.962 ± 0.029	21		16
A24U	0.878 ± 0.057	21		0
A37G	0.861 ± 0.032	15		32
G39U	0.915 ± 0.033	30		0
A46U	0.910 ± 0.042	21		8
A54G	0.823 ± 0.058	21		49
C57U	0.808 ± 0.052	15		4
G75U	0.802 ± 0.061	18		47
A83G	0.927 ± 0.055	10		53
A84G	0.866 ± 0.037	35		10
C91U	0.913 ± 0.033	32		0
C100G	0	30		48
C106U	0.896 ± 0.051	24		37
C127U	0	30		53
U131C	0.997 ± 0.023	15		0
A135G	0.971 ± 0.033	27		7
G138U	0.985 ± 0.023	24		2
G142A	0.873 ± 0.038	27		32
A16G/C23G	0.999 ± 0.024	24	0.117 ± 0.089	40
A16G/C100G	0.902 ± 0.042	27	0.903 ± 0.048	33
A16G/C127U	0	30	0	53
A16G/A135G	1.012 ± 0.020	21	0.120 ± 0.089	7
C18A/C100G	0	30	0	48
C23G/A24U	0.932 ± 0.034	21	0.088 ± 0.120	10
C23G/A54G	1.040 ± 0.005	3	0.250 ± 0.091	57
C23G/142A	0	30	−0.839 ± 0.062	40
A24U/C106U	1.031 ± 0.056	21	0.244 ± 0.159	37
A37G/C106U	0.899 ± 0.024	21	0.127 ± 0.102	18
A37G/A135G	0.956 ± 0.004	30	0.120 ± 0.070	7
G39U/C91U	0.901 ± 0.032	27	0.066 ± 0.098	0
A46U/A135G	0.900 ± 0.013	32	0.017 ± 0.090	15
C57U/G75U	0.906 ± 0.039	30	0.259 ± 0.135	8
C57U/A83G	0.823 ± 0.052	9	0.074 ± 0.150	45
C57U/C127U	0.949 ± 0.037	27	0.949 ± 0.043	35
A83G/C100G	1.019 ± 0.019	18	1.019 ± 0.026	53
A84G/G142A	0.999 ± 0.014	24	0.244 ± 0.086	32
C91U/U131C	1.016 ± 0.015	30	0.106 ± 0.075	0
C91U/A135G	0.833 ± 0.007	3	−0.053 ± 0.074	7
C100G/C106U	0.751 ± 0.035	11	0.751 ± 0.040	48
C100G/G138U	0.635 ± 0.002	3	0.635 ± 0.006	33
C127U/A135G	0.816 ± 0.077	6	0.817 ± 0.082	7
U131C/G142A	0.705 ± 0.048	6	−0.166 ± 0.111	20
A135G/G138U	0	10	−0.957 ± 0.055	9

**Table 2:** GLM analysis of the fitness data

Effect	*LRT*	d.f.	*P*	*η*_*P*_^2^
Intercept (*μ*)	7243.749	1	<0.001	0.986
Mutant genotype (*M*)	5474.979	45	<0.001	0.936
Experimental block (*B*)	2572.850	1	<0.001	0.358
Interaction (*M* × *B*)	3142.981	45	<0.001	0.184
Plant replicate (*P*(*M* × *B*))	4385.585	220	<0.001	0.994

*LRT* is the value of the likelihood ratio test, *P* is its corresponding significance level and the *η*_*P*_^2^ statistic represents the proportion of the total variability attributable to a each factor in the model

### Properties of the DMFE for the 5’ UTR

**Fig. 2:**
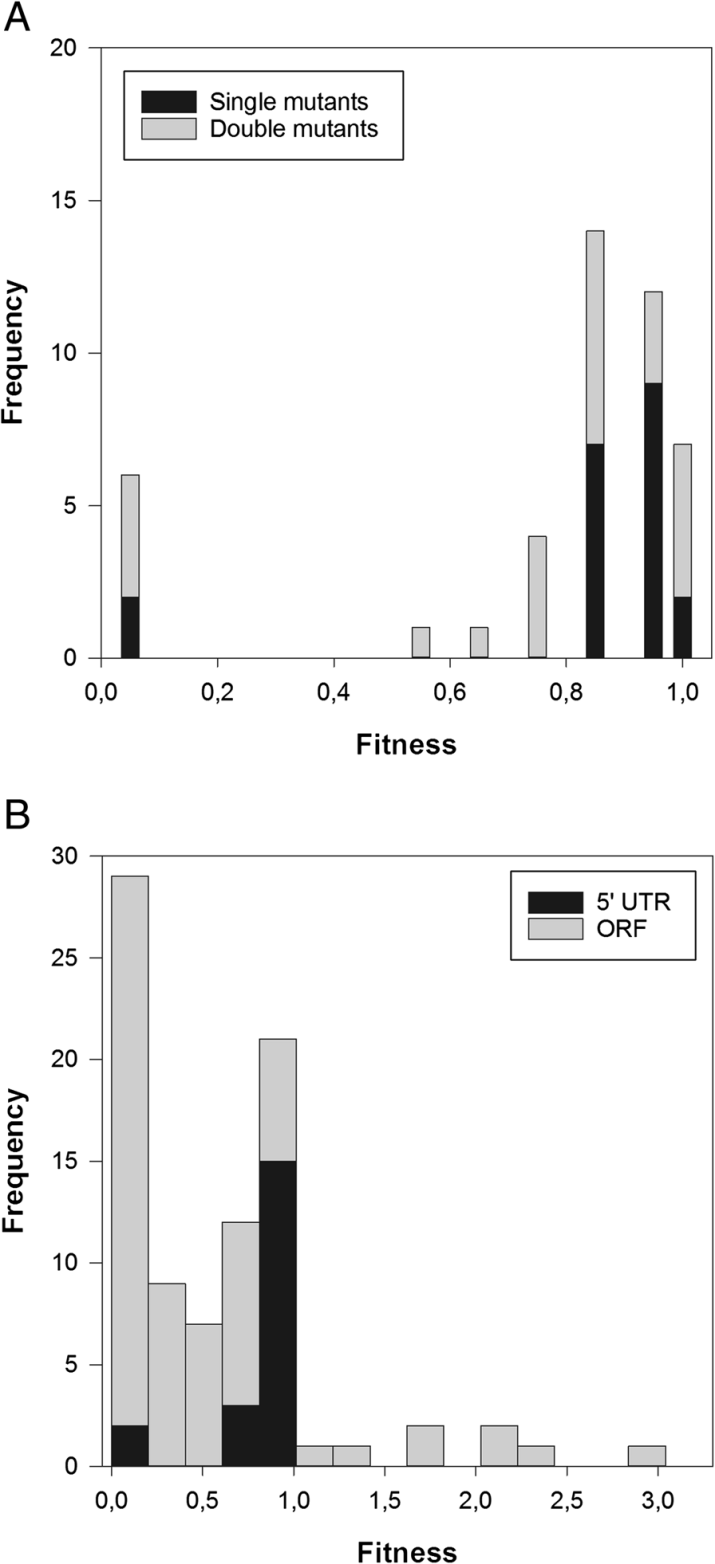
**a** DMFEs measured for single and double mutants of TEV 5’ UTR. **b** Comparison of the DMFE of single mutations affecting TEV 5’ UTR and ORF. ORF data are taken from Carrasco et al. [19]. Notice that bar counts are juxtaposed

After proving the existence of significant differences in fitness among 5’ UTR mutants, we proceed to calculate some summary statistics of the DMFE. Figure 2a shows the histogram of fitness values for single and double mutants. For the sake of analyzing the DMFE, only single mutants are relevant. The distribution has a mean value of 0.793 ± 0.048, which is smaller than the median value (0.905), thus indicating an asymmetric distribution dominated by deleterious mutational effects. Indeed, the distribution was significantly skewed towards small fitness values (−1.958 ± 0.350; *t*_19_ = −5.594, *P* < 0.001); i.e., the left tail of fitness values smaller than the mean has more weight that the tail of greater-than-the-mean fitness values.

Next, we sought to evaluate which individual mutations significantly affected TEV fitness. To do so, we compared the empirical fitness values obtained for each mutant to the values measured for the WT. Pairwise comparisons on the GLM model fitted in the previous section, with a sequential Holm-Bonferroni correction for multiple tests of the same null hypothesis, were employed to assess the significance of each mutant effect. Only two out of the 20 single mutants generated had no significant effect on TEV fitness (i.e., *W* = 1). These two mutations are C23G, located in the 5’ unstructured region, and U131C, located in the large bulge of SL3 (Fig. 1b). Significant effects ranged from the extreme case of lethal mutations C100G and C127U (i.e., *W* = 0) to the 7.49 % significant beneficial effect associated to mutation A24U. Lethal mutation C100G is located in the base of SL2 and lethal mutation C127U is involved in the larger bulge of SL3 (Fig. 1b), whereas beneficial mutation A24U is located in the 5’ unstructured region.

### No significant differences in fitness effects among mutations affecting paired and unpaired sites

Next, we sought to evaluate whether the effect of point mutations affecting paired and unpaired residues was similar. A priori, one may hypothesize that altering paired residues may have a stronger effect on fitness, as they may alter the configuration of SLs. Following the same logic, mutations affecting unpaired residues may have a weaker effect because they do not alter SLs. An alternative hypothesis being that both types of mutations are of similar fitness effect because both paired and unpaired nucleotides are essential for interactions of the IRES with host proteins and other RNAs. Twelve mutations in our collection of single mutants affect unpaired nucleotides, having an average fitness of 0.857 ± 0.080. Eight mutations affect paired nucleotides, in this case having an average fitness of 0.771 ± 0.112. However, the −8.60 % difference between paired and unpaired sites was not statistically significant (two samples *t*-test with equal variances, *t*_18_ = 0.642, *P* = 0.529), although the power associated to this test is really low (1 − *β* = 0.093). Therefore, we have not enough statistical power as to convincingly reject the hypothesis of equal effects even if the alternative hypothesis of smaller effects for unpaired sites was actually true.

### Comparing the effect of mutations affecting the 5’ UTR and the ORF

In a previous study, Carrasco et al. [19] evaluated the effect of point mutations in the TEV ORF using a similar experimental approach. They found that the average fitness of genotypes carrying a point mutation affecting the ORF was 0.510 ± 0.080. This value is significantly smaller than the average fitness estimated here for the 5’ UTR (Fig. 2b; *U*-test, *P* < 0.001). However, it is important to notice here that the fraction of lethal mutations affecting the ORF was larger than for the 5’ UTR (Fisher’s exact test, *P* = 0.036). Even after removing lethal mutations in both datasets, on average, mutations affecting the ORF had a stronger negative impact in TEV fitness (0.863 ± 0.103) than those affecting the 5’ UTR (*U*-test, *P* = 0.004).

The collection of mutations in Carrasco et al. [19] includes both synonymous and nonsynonymous mutations. Strictly speaking, mutations in the 5’ UTR would qualify as synonymous since they involve no amino acid change. Therefore, it is worth comparing the average effect of a synonymous mutation affecting the ORF (0.859 ± 0.248) with the effect of mutations at the 5’ UTR. In this case, no significant difference can be detected (*U*-test, *P* = 0.224).

### Descriptive statistics for the double mutants

The overall shape of the distribution of fitness values for the double mutants (Table 1; Fig. 2a) differs from what shall be expected for a Normal distribution (Kolmogorov-Smirnov test, *P* < 0.001). The observed distribution is slightly leptokurtic, with a kurtosis coefficient of 1.284 ± 0.902 that is significantly smaller than the expected value of three for the Normal distribution (*t*_24_ = 1.424, 1-tailed *P* = 0.034), meaning that it is more peaked than the Normal distribution. The distribution is centered on a mean value of 0.761 ± 0.071, a value smaller than the median (0.902), thus suggesting a certain degree of asymmetry. Indeed, the skewness value estimated (−1.649 ± 0.464) was significantly different from zero, the expected value for a Normal distribution (*t*_24_ = 3.554, *P* = 0.002).

As in our analysis of single mutations, now we sought to determine which double mutants had fitness values that significantly deviated from the WT. Using again the pairwise comparisons on the GLM model fitted above, with a sequential Holm-Bonferroni correction for multiple tests of the same null hypothesis, we found that seven double mutants had no significant fitness effects compared to the WT virus (A16G/C23G, A16G/A135G, A24U/C106U, A83G/C100G, A84G/G142A, C23G/A24U, and C91U/U131C). All other pairs of mutations had significant deleterious effects ranging between lethality (A16G/C127U, C18A/C100G and A135G/G138U) and −4.40 % (A37G/A135G). Interestingly, these extreme cases always involve mutations in the SL2 or SL3 (Fig. 1b).

### Epistasis analysis: statistics and types

**Fig. 3:**
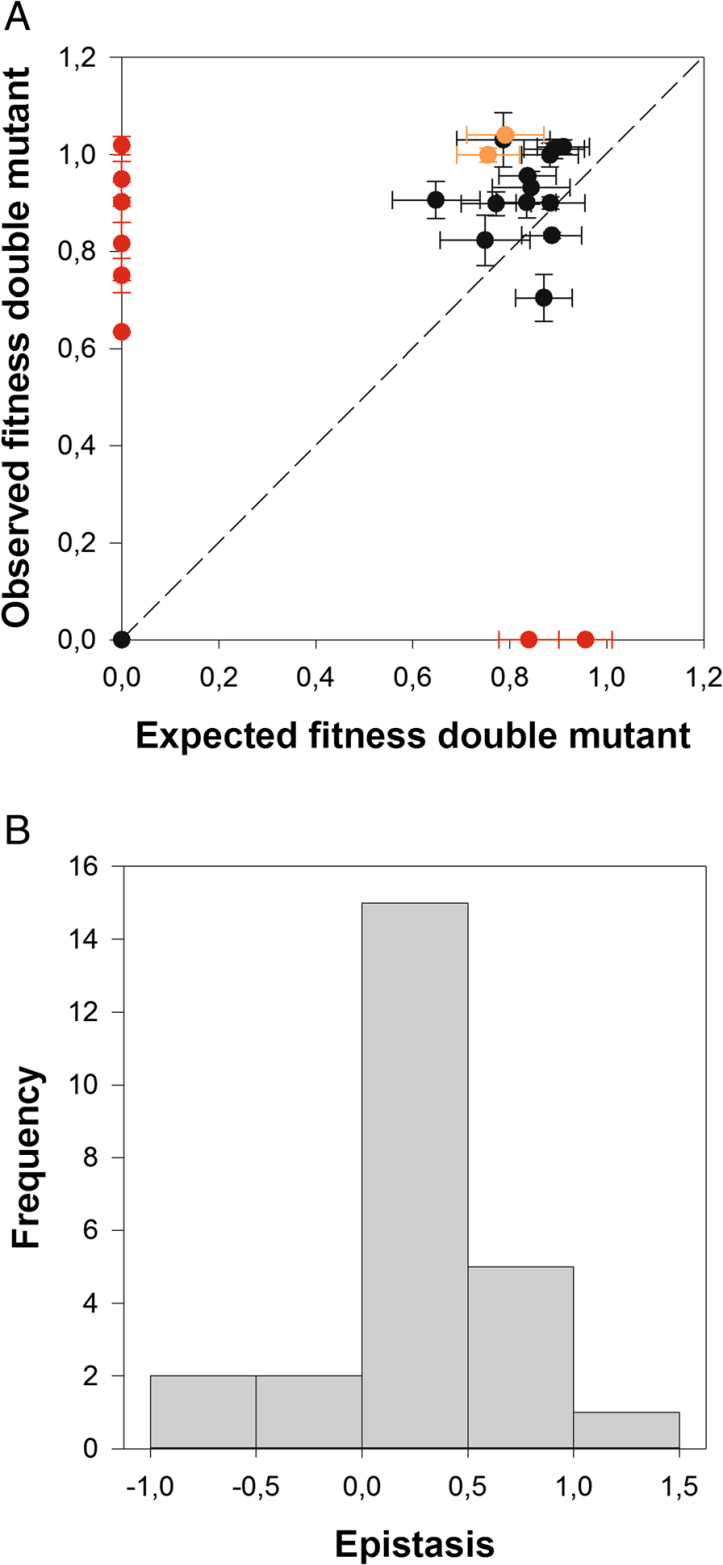
**a** Relationship between observed and expected multiplicative fitness for 25 TEV genotypes carrying pairs of nucleotide substitutions in the 5’ UTR. The dashed line represents the null hypothesis of multiplicative fitness effects. Deviations from this line arise as a consequence of the existence of epistatic fitness effects. In orange, pairs that do not deviate from the null expectation if the Holm-Bonferroni sequential correction is applied. In red, pairs that do significantly deviate even after applying the Holm-Bonferroni sequential correction level for the overall significance level. **b** Distribution of epistasis, *ε*. Epistasis was computed as the difference between the observed fitness of the double mutant (*W*_00_ *W*_*ij*_) and the value expected from subtracting the effects of each single mutant from the WT value (*W*_*i*0_ *W*_0*j*_)

Figure 3a shows the relationship between observed and expected fitness values for the set of 25 double mutant genotypes synthetized for this study. The dashed line represents the null hypothesis of non-epistatic fitness effects. Table 1 shows the estimated epistasis coefficients. The observed fitness values of 10 double mutant genotypes significantly depart from this null expectation (red and orange symbols) according to 1-sample *t*-tests, although only eight cases (red symbols) remained significant after applying the more stringent Holm-Bonferroni criterion. Focusing in the latter cases, two were cases of extreme negative epistasis (i.e., synthetic lethals) in which two mutations that were independently viable, generated a lethal genotype when combined. Interestingly, both mutations in synthetic lethal A135G/G138U affected the same strand of the basal SL3 stem (Fig. 1b). The other six significant cases correspond to the opposite situation: pairs that involved a mutation by itself lethal but whose effect was compensated by the second mutation (i.e.*,* compensatory mutations). In four of these cases, mutation C100G (in the base of stem SL2) was involved. Therefore, we found variability in the sign and strength of epistasis in the 5’ UTR.

Figure 3b illustrates the distribution of epistasis parameters for all pairs of point mutations analyzed. The distribution is unimodal with an average value of 0.196 ± 0.095. The observed distribution is symmetrical (non significant skewness: −0.377 ± 0.464, *t*_24_ = 0.813, *P* = 0.5281) and slightly leptokurtic (kurtosis: 1.048 ± 0.902, *t*_24_ = 2.164, *P* = 0.043), as expected for a Normal distribution. As shown in Table 1, there are four estimates of epistasis that are negative, two that are zero and 19 that are positive. Ignoring the two cases of no epistasis, a one-sample Binomial test shows that there is a significant enrichment in cases of positive epistasis (*P* = 0.001). This dominance of cases of positive epistasis is consistent with what has been described for the ORF of TEV [29].

Applying Poelwijk et al. [53] criteria, we found that from the eight pairs of mutations that showed significant epistatic interactions, five were of the ME type (C23G/G142A, C100G/C106U, C100G/G138U, C127U/A135G, and A135G/G138U), one of the SE type (A16G/C100G) and two of RSE type (C57U/C127U and A83G/C100G). These results illustrate several points. First, that the same mutation (e.g., C100G) can be involved in all types of epistatic interactions. Second, compensatory epistasis is generally assumed to be of the SE or RSE types, such as the deleterious effect of one mutation is compensated by the presence of a second one. However, with our reduced sample size, this assumption is not supported: half of the cases of compensation are associated to SE or RSE while the other half corresponds to cases of ME.

### Correlation between the size of perturbation in the in vitro structure and the magnitude of in vivo fitness effect

**Fig. 4:**
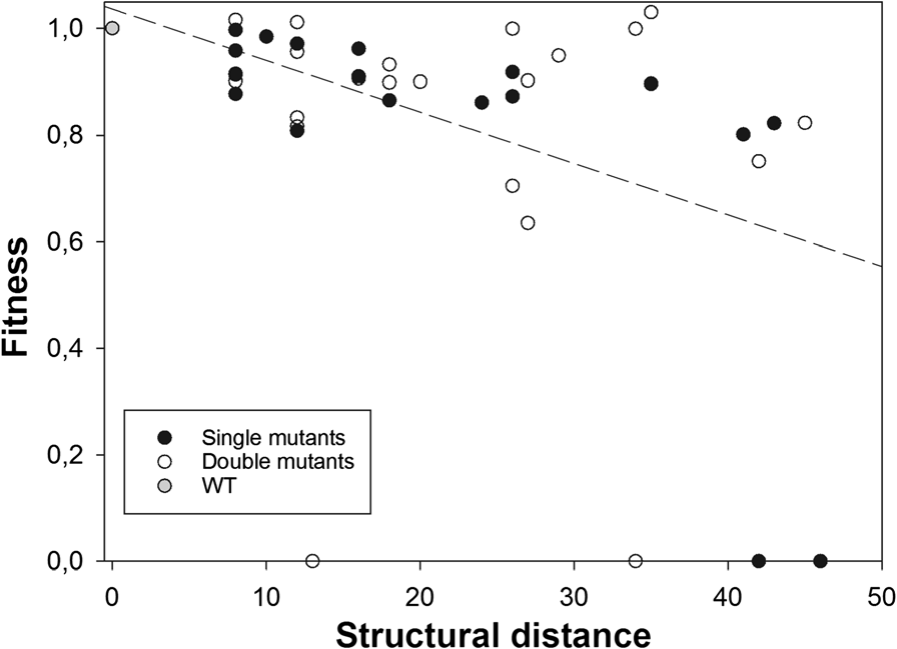
Relationship between the size of the structural perturbation induced by mutations and fitness. The regression line is included only to highlight the existence of a negative association

Finally, we sought to test whether stronger deleterious mutational effects were associated to larger perturbations in the folding of the 5’ UTR, whereas minor perturbations had little fitness effects. Figure 4 shows the relationship between in vivo fitness and the size of the structural perturbation *d*_*ij*_, computed *in silico* as the Hamming distance between the structure shown in Fig. 1b and the MFESS obtained for each mutant. A partial correlation test, controlling for the number of mutations, founds a significant negative association between these two traits (*r* = −0.327, 43 d.f., *P* = 0.028), meaning that the stronger the perturbation induced in the RNA folding, the smaller the fitness of the mutant genotype, thus linking structure conservation with viral fitness.

## Discussion

A growing body of evidence supports the view that regulatory evolution, that is, the evolution of the mechanisms controlling when and where genes are expressed, is fundamental to understand the modular organization of genomes, their functional diversification, the origin of novel phenotypic traits in absence of major protein differences, and even speciation [55–57]. Mutations arising within *cis*-regulatory elements can generate variation in downstream genes expression by changing the way transcription factors or ribosomes bind. Tighter or looser bindings may lead to up- or down-regulated transcription. Comparative genomic and transcriptomic studies have provided insights into the evolutionary pressures that shape gene expression levels and have highlighted the relative importance of evolutionary changes in regulatory sequences [57]. For obvious reasons, eukaryotes have been the main focus of these studies, and less information is available about the potential role of regulatory evolution in the apparently simplest viruses. However, this simplicity makes viruses ideal tools for analyzing the effect of mutations in regulatory sequences in the expression of regulated genes and, hence, in fitness. In an attempt to cover this gap, here we provide the results of an experiment in which the evolutionary role of mutations in the 5’ UTR regulatory sequence of TEV, a prototypical member of the picorna-like family, is under investigation. In this paper, we quantitatively describe the statistical properties of the distribution of mutational fitness effects and the spectrum of epistasis for fitness for random mutations affecting this regulatory region.

Several previous studies have characterized the DMFE [17–20, 22, 23] and epistatic interactions [28, 29] among random pairs of mutations for RNA and small DNA viruses, though most of them focused on mutations affecting coding sequences. To the extent of our knowledge, only two studies specifically focused on non-coding regulatory regions [30, 31]. To bridge this gap, we have performed a systematic characterization of the DMFE and epistasis for the 5’ UTR of TEV, a virus that has become a model for experimental virus evolution. As a preliminary step in our analyses, we decided to experimentally evaluate the secondary structure of TEV 5’ UTR in vitro using the SHAPE technique. The TEV 5’ UTR secondary structure presented here substantially differs from the one previously described from deletion analyses and diverse enzymatic and chemical probing studies [35]. In this previous study, the putative folding also contained three stem-loops, but involving nucleotides 38–60, 78–97 and 106–120, which differ from the SL1-SL3 described here. A second major difference between our structure and this previous one is that the latter also involved three pseudoknots, one of them dubbed as essential for full cap-independent translation [35]. However, inconsistent with this supposed essentiality, mutations introduced in this region had no major effects and compensatory mutations did not restore translation activity [35]. Interestingly, the nucleotides described as involved in this pseudoknot (47–51 and 68–72) are forming part of the SL1 predicted by our SHAPE results. In any case, based on their common simplicity, potyvirus 5’ UTRs have been hypothesized to lack extensive secondary structure and function in translation enhancement with the participation of a stretch of seven nucleotides of the 18S rRNA, highly conserved among all eukaryotes, complementary to nucleotides 57–75 of TEV 5’ UTR [6, 35].

It is generally assumed that the presence of SLs in the 5’ UTR of potyviruses are essential for the cap-independent regulatory IRES role of this non-coding region [35, 36]. This being the case, one should expect mutations affecting the folding of the three SLs to have a stronger effect than mutations not affecting their fold. However, our results proved this expectation to be *naïve*. We have observed that mutations affecting paired and unpaired residues are, on average, of the same effect. There are several possible interpretations for this lack of differences. First, a trivial, yet frustrating, explanation is that our *in silico* and SHAPE in vitro structure does not reflect at all the real in vivo configuration as it fails to take into account how proteins (both cellular and viral) and other RNAs may interact and condition the 5’ UTR folding and, therefore, we are not really contrasting paired to unpaired residues. Assuming that our structure is of biological relevance, a second possible explanation is that not only positions directly involved in the maintenance of the SLs are important for the IRES, but also unpaired positions may be involved either in the establishment of the proper RNA-protein interactions or in potential pseudoknots between terminal loops and unpaired regions, specially the 5’-proximal 75 nucleotides [35]. A third more tantalizing possibility is that selection for robustness had operated at different levels. First, natural selection may have shaped TEV 5’ UTR folding to be robust to mutational changes and, therefore, most mutations do not have an strong effect on RNA folding simply because they have no major effect on the structure [44]. Indeed, this is compatible with TEV 5’ UTR being considerably shorter in length, less structured and contains no upstream AUG triplets, compared to other members of the picorna-like superfamily. Contrasting with our results, the only other study that specifically addressed differences in mutational effects on paired and unpaired regulatory regions for an RNA virus was done for the U5-IR SL of RSV, and found that mutations affecting the stem had a 1.6-fold more negative fitness effect than mutations affecting the loop [30]. Second, the host translational machinery has evolved to be robust enough so any RNA sequence/structure that meets some relatively simple requirements is readily translated. The fact that viruses have found a completely cap-independent mechanism for doing so emphasizes this possibility.

Interestingly, the magnitude of fitness effects measured in vivo and the size of the perturbation induced in the *in silico* RNA folding by mutations are positively correlated (Fig. 4). This means that the stronger the structural perturbation induced by mutations, the lower the fitness of the mutant virus was. *In silico* RNA folding is being used by many researchers as a good computational model of the genotype-to-phenotype map (e.g., [44, 45, 58–60]). Alas, how well this approximation would represent a real biological system was unclear, as in many instances empirical evidences linking *in silico* structures with biological functions were missing. Our observation provides evidence that the approach may be valid for studies performed with real (not simulated) RNA sequences, at least for sequences folding in simple RNA structures, as those shown here for the TEV 5’ UTR.

Another interesting observation we made here is that the average fitness effect of a random mutation affecting the 5’ UTR is significantly weaker than the effect measured for another random mutation affecting the ORF. Indeed, the effect of a random mutation affecting the 5’ UTR is equivalent to the effect of a synonymous random mutation affecting the ORF. Unfortunately, it is hard to assess the generality of this observation owed to the lack of information on mutational fitness effects on viral non-coding regulatory regions. The average effect of single nucleotide substitutions in coding regions is close to −10 % for different RNA and ssDNA viruses [26], after accounting for differences in the number of generations and without considering lethal mutations, which in every case represented a substantial fraction of total mutations. After transforming our estimates into the same common scale following the method described by Sanjuán et al. [26], the average effect of mutations in TEV 5’ UTR is close to −3 %. The fitness effects measured for random mutations affecting the DNA binding sites of the transcriptional promoter of HIV-1 were even weaker, −0.4 % [31]. In sharp contrast, the fitness values reported for mutations affecting RSV U5-IR [30], also expressed in the same numerical scale, are substantially more deleterious (−87 % for paired and −55 % for unpaired sites) than the above across-virus species average of −10 %. Therefore, with the precaution due to the small sample size and differences in methodologies, it seems that mutations affecting viral coding sequences are more homogeneous among viral species than mutations affecting non-coding regulatory sequences, which tend to widely vary, as expected for the tremendous differences in transcription regulation of these viruses. It is fair mentioning that there is another potential misleading factor in the comparison between mutations in TEV 5’ UTR and in the ORF: mutations in Carrasco et al. [19] were selected at random, while mutations here were selected to cover the entire range of effects on the *in silico* secondary structure. Since the *in silico* predicted and the in vitro estimated structures are not identical, the selection of mutations based on the *in silico* effect on structure is quasi random in the in vitro structure, and most likely mostly random in the in vivo structure.

A general observation for compacted RNA virus genomes is that positive epistasis (also known as diminishing returns or antagonistic epistasis) is the predominant type of interactions among random pairs of mutations affecting coding sequences [61]. This means that the combined effect of two mutations is less than expected from simply multiplying the independent effects of each single mutant. Again, data on epistatic interactions among pairs of mutations affecting non-coding regulatory regions are scarce for RNA viruses. Epistatic interactions among pairs of mutations affecting the RSV U5-IR were positive [30] and of similar value for pairs affecting the stem or the loop. By contrast, no significant epistasis was found among pairs of mutations affecting the DNA binding sites of the transcriptional promoter of HIV-1 [31]. Here we found variability in the strength and sign of epistatic interactions for TEV 5’ UTR: most mutations had independent effects, but pairs exist showing extreme positive (compensatory) or negative epistasis (synthetic lethals), but with a significant enrichment in cases of positive epistasis. In the particular case of TEV, random pairs of mutations affecting the ORF also show variability in the strength and sign of epistatic interactions, with most pairs showing multiplicative effects and 38 % of pairs showing positive epistasis [29].

A very important question from an evolutionary perspective is the actual nature of epistasis, since the existence of multiple adaptive peaks in the fitness landscape, and their accessibility depends on whether epistasis are of the magnitude, of the sign or of the reciprocal sign types [53]. ME means that the landscape is still smooth and dominated by a single peak, although its global curvature departs from the null multiplicative model (i.e., it may be concave or convex instead of flat). SE, and especially RSE create ruggedness in the landscape: multiple peaks exist, which in the case of RSE are not accessible from one to another unless crossing a low fitness valley. In the case of pairs of mutations affecting TEV ORF, most of significant epistatic pairs were of the RSE type [29]. This observation has been extended here for pairs of mutations affecting TEV 5’ UTR (two cases of RSE among eight significant cases of epistasis). The pervasiveness of RSE in TEV genome suggests that its adaptive fitness landscape is highly rugged, and thus the virus may get easily trapped in local adaptive peaks.

## Conclusions

In conclusion, we found evidence that mutational fitness effects on the short 5’ UTR regulatory sequence of TEV are weaker than those affecting its long ORF. Why is this so? At this stage we can only speculate, but a certainly interesting hypothesis to be tested is that it may be due to differences in the selective pressures that have modeled each region during the past evolution of this virus. Perhaps selection for mutational robustness was stronger in the 5’ UTR than in the coding sequence because it has to ensure the recruitment of the host translational machinery. In agreement with the robustness hypothesis, we also found that epistasis among pairs of mutations on the 5’ UTR ranged between the extreme cases of synthetic lethal and compensatory, with an excess of positive epistasis.

## Availability of supporting data

Fitness data and epistasis computations are available in LabArchives data repository with doi: 10.6070/H4KP805W.

## Abbreviations

B: experimental block
CIRE: cap-independent regulatory element
DMFE: distribution of mutational fitness effects
GLM: general linear model
HEPES: 4-(2-hydroxyethyl)-1-piperazineethanesulfonic acid
HIV-1: *Human immunodeficiency virus* type 1
IRES: internal ribosomal entry site
LRT: likelihood ratio test
M: mutant genotype
ME: magnitude epistasis
MFESS: minimum free energy secondary structure
NMIA: N-methylisatoic anhydride
P: plant replicate
RNA: ribonucleic acid
RSE: reciprocal sign epistasis
RSV: *Rous sarcoma virus*
SE: sign epistasis
SEM: standard error of the mean
SHAPE: selective 2’-hydroxyl acylation analyzed by primer extension
TEV: *Tobacco etch virus*
UTR: untranslated region
VPg: genome-linked viral protein
*W*: relative fitness
